# Genome Sequences of *Vibrio maerlii* sp. nov. and *Vibrio rhodolitus* sp. nov., Isolated from Rhodoliths

**DOI:** 10.1128/MRA.01039-18

**Published:** 2018-11-15

**Authors:** Camila S. F. Silva, Juline M. Walter, Maria S. Nobrega, Gabriela Calegario, Luciana R. Appolinario, Luciana Leomil, Giselle Cavalcanti, Bruno S. Silva, Gizele D. Garcia, Diogo Tschoeke, Jean Swings, Fabiano L. Thompson, Cristiane C. Thompson

**Affiliations:** a Institute of Biologia, Federal University of Rio de Janeiro, Rio de Janeiro, Brazil; b Departamento de Ensino de Graduação, Universidade Federal do Rio de Janeiro–Campus UFRJ–Macaé Professor Aloisio Teixeira, Macaé, Rio de Janeiro, Brazil; c SAGE-COPPE, Federal University of Rio de Janeiro, Rio de Janeiro, Brazil; Iowa State University

## Abstract

We report here the genome sequences of the novel isolates G62^T^ and G98^T^ from rhodoliths. The nearly complete genomes consisted of 4.7 Mbp (4,233 coding sequences [CDS]) for G62^T^ and 4.5 Mbp (4,085 CDS) for G98^T^. Genomic taxonomy places these new genomes into 2 new species.

## Abstract (precis)

We report here the genome sequences of the novel isolates G62^T^ and G98^T^ from rhodoliths. The nearly complete genomes consisted of 4.7 Mbp (4,233 coding sequences [CDS]) for G62^T^ and 4.5 Mbp (4,085 CDS) for G98^T^.

## ANNOUNCEMENT

The genus Vibrio and 6 other genera (Aliivibrio, Echinimonas, Enterovibrio, Grimontia, Photobacterium, and Salinivibrio) form the family *Vibrionaceae* (1). These bacteria are Gram-negative fermentative, halophilic, mesophilic, chemoorganotrophic, and ubiquitous in the marine environment (2).

The aim of the present study was to determine the genome sequences of the novel isolates G62^T^ and G98^T^. These isolates were obtained from rhodoliths at a 43-m depth (sinkhole, Abrolhos Bank, Brazil, 17.81399°S, 38.24306°W and 17.81330°S, 38.23744°W) in 2010. Samples were cultured in marine agar and incubated at 30°C for 48 h. Genomic DNA was extracted with the NucleoSpin tissue kit (Macherey-Nagel GmbH & Co. KG) and used for 300-bp paired-end library preparation with the Nextera XT DNA sample preparation kit and sequencing on the MiSeq platform (Illumina, San Diego, CA, USA) (3). The sequences obtained were preprocessed with PRINSEQ software to remove reads smaller than 35 bp and low-score sequences (Phred < 30) (4). Sequence reads were assembled with A5-miseq software v. 20160825 (5). A second assembly was performed with CAP3 software (6). The gene prediction and functional annotation were performed with the Rapid Annotation using Subsystem Technology program (7).

The reads from G62^T^ were assembled in 49 contigs (*N*_50_, 382,658 bp). The estimated genome size is 4,758,035 bp (G+C content, 44.5%) with a coverage of 108-fold. In total, 4,233 coding sequences (CDS), 143 RNAs, 106 tRNAs, and 26 rRNAs were identified. G98^T^ reads were assembled in 62 contigs (*N*_50_, 161,850 bp). The estimated genome size is 4,559,723 bp (G+C content, 45.2%) with a coverage of 94-fold. In total, 4,085 CDS, 143 RNAs, 108 tRNAs, and 24 rRNAs were identified.

The novel isolates G62^T^ and G98^T^ had less than a 95% average amino acid identity/average nucleotide identity (AAI/ANI) and less than a 70% genome-to-genome distance (GGDH) from their closest neighbors (V. atypicus and V. tubiashii and V. ponticus and V. furnissii, respectively). The cutoffs used for the delimitation of Vibrio species are more than 95% AAI and more than 70% GGDH, and so we conclude that the 2 novel isolates belong to novel species of the genus (8–11), designated V. maerlii G62^T^ sp. nov. and V. rhodolitus G98^T^ sp. nov. Their cells are motile, and growth occurs between 12 and 35°C in the presence of 1.0 to 3.0% NaCl. Optimum bacterial growth occurs between 27 and 30°C in the presence of 3.0% NaCl. Colonies are cream colored and circular with the entire margin on marine agar. Useful *in silico* phenotypic features to differentiate the novel species from their closest neighbors include sucrose, indole, d-mannitol, cellobiose, and d-mannose (Table 1).

**TABLE 1:** *In silico* phenotypic characterization distinguishing *Vibrio maerlii* sp. nov. strain G62^T^ and *Vibrio rhodolitus* sp. nov. strain G98^T^ from closely related *Vibrio* species^*a*^

Property	*V. maerlii* G62^T^	*V. rhodolitus* G98^T^	*V. fluvialis* ATCC 33809^T^	V. furnissii NCTC 11218^T^	*V. parahemolyticus* RIMD 2210633^T^	*V. ponticus* CECT 5869^T^	*V. scophthalmi* VS-12	*V. tasmaniensis* LGP32	*V. tubiashii* ATCC 19109^T^	*V. xuii* DSM 17185^T^
l-Arabinose	−	−	+	+	+	−	−	−	−	−
Sucrose	+	−	+	+	−	−	−	−	+	−
Ornithine	−	−	−	−	+	−	−	−	−	−
Vogues	−	−	−	−	−	−	−	−	−	−
Galactose	−	−	+	±	+	−	−	−	−	−
Cellobiose	−	+	±	−	−	+	+	−	−	+
d-Mannitol	+	−	−	+	+	+	+	−	+	+
Arginine	−	−	+	+	−	−	−	+	−	+
Trehalose	−	−	+	+	+	−	−	+	−	+
d-Sorbitol	−	−	−	−	−	−	−	−	−	−
Indole	+	+	±	−	+	+	+	+	+	+
m-Inositol	−	−	−	−	−	−	−	−	−	−
d-Mannose	−	+	±	+	+	+	+	+	−	+

a Closely related *Vibrio* species (RefSeq assembly accession number) include the following: *V. fluvialis* ATCC 33809^T^ (GCF_001558415), *V. furnissii* NCTC 11218^T^ (GCF_000176175), *V. parahaemolyticus* RIMD 2210633^T^ (GCF_000196095), *V. ponticus* CECT 5869^T^ (GCF_001939685), *V. scophthalmi* VS-12 (GCF_001685465), *V. tasmaniensis* LGP32 (GCF_000091465), *V. tubiashii* ATCC 19109^T^ (GCF_000772105), and *V. xuii* DSM 17185^T^ (GCF_001274855). +, present; −, absent; ±, variable.

### Description of Vibrio maerlii sp. nov.

Vibrio maerlii (ma.er′.li.i. L. n. maerlii, referring to the Breton word “maërl” = rhodolith). The estimated genome size is 4.7 Mbp, and it has a G+C content of 45.2%. G62^T^ is positive for sucrose, indole, and d-mannitol based on an *in silico* phenotype (12).

### Description of Vibrio rhodolitus sp. nov.

Vibrio rhodolitus (rhod.o.li′.tus. L. masc. rhodoliths, referring to the isolation source, the rhodolith structures dominated by free-living coralline algae). The estimated genome size is 4.5 Mbp, and it has a G+C content of 44.5%. G98^T^ is positive for cellobiose, indole, and d-mannose based on an *in silico* phenotype (12).

### Data availability.

V. maerlii G62^T^ and V. rhodoliths G98^T^ are deposited in the Bacteria Collection of Environmental and Health (CBAS) at the Oswaldo Cruz Institute (IOC), Oswaldo Cruz Foundation (FIOCRUZ) in Rio de Janeiro, Brazil (http://cbas.fiocruz.br/), under the accession numbers CBAS 711^T^ and CBAS 710^T^, and in the Collection of Aquatic Microorganisms (CAIM) in Mazatlán, Sinaloa, Mexico (http://www.ciad.mx/caim/CAIM.html), under the accession numbers CAIM 1940^T^ and CAIM 1941^T^, respectively.

The whole-genome shotgun projects for V. maerlii G62^T^ (= CBAS 711^T^ = CAIM 1940^T^) and V. rhodoliths G98^T^ (= CBAS 710^T^ = CAIM 1941^T^) have been deposited in DDBJ/EMBL/GenBank under accession numbers QRHC00000000 and QLYZ00000000, respectively. The BioProject accession numbers are PRJNA476538 and PRJNA476543, respectively.
